# Evaluating the Adaptive Fitness of Circadian Clocks and their Evolution

**DOI:** 10.1177/07487304231219206

**Published:** 2024-01-07

**Authors:** Maria Luísa Jabbur, Chitrang Dani, Kamiel Spoelstra, Antony N. Dodd, Carl Hirschie Johnson

**Affiliations:** * Department of Biological Sciences, Vanderbilt University, Nashville, Tennessee, USA; † Department of Animal Ecology, Netherlands Institute of Ecology (NIOO-KNAW), Wageningen, The Netherlands; ‡ Department of Cell and Developmental Biology, John Innes Centre, Norwich, UK

**Keywords:** circadian, chronobiology, evolution, competition assay, cyanobacteria, fitness, phase angle

## Abstract

Surely most chronobiologists believe circadian clocks are an adaptation of organisms that enhances fitness, but are we certain that this focus of our research effort really confers a fitness advantage? What is the evidence, and how do we evaluate it? What are the best criteria? These questions are the topic of this review. In addition, we will discuss selective pressures that might have led to the historical evolution of circadian systems while considering the intriguing question of whether the ongoing climate change is modulating these selective pressures so that the clock is still evolving.

## “Fitness” and “adaptation”

Given the strong 24-h fluctuations in natural light and concurrent selection pressure for temporal schedules, it is perhaps not surprising that circadian clocks are widespread. But “widespread” is not synonymous with “adaptive.” Have we chronobiologists been guilty of “adaptive storytelling?” Is there definitive support for an adaptive value of circadian clocks? As an entry point to address these questions, allow us to define two key terms, “fitness” and “adaptation.” The fitness of a genotype is the average per capita lifetime contribution of individuals of that genotype to the population after one or more generations (Futuyma, 1998). Fitness is a measure of reproductive success and the passing on of genes. Fitness may be influenced by longevity, survival, growth, development, fecundity, and other factors, but while these factors represent components of fitness, they are not direct measures of reproductive fitness. For example, consider an illustrative fable provided to one of us (C.H.J.) by Michael Menaker in an introductory biology class. A mighty male lion might dominate his pride and survive to a ripe old age, but if he is sterile, his fitness is zero. Rather, it might be the wimpy but fertile lion who lurks in the shadows and clandestinely inseminates temporarily unguarded lionesses who will pass his genes to the next generation. The stealthy lion might die an early death (possibly after being caught *flagrante delicto* by the mighty lion), but his reproductive fitness is greater than that of the long-lived mighty lion. This fable was not meant to be an accurate statement of lion behavior, but Menaker meant it to capture a key point about evolution that he liked to underscore with the tautology, “the most efficient reproducers reproduce most efficiently.”

The second key term is adaptation. This term is used in two different ways by evolutionary biologists. An adaptation refers to an aspect of the phenotype that is the product of evolution by natural selection in a particular environmental context and represents a solution to some challenge presented by the environment. In this sense, an adaptation is a trait of an organism that enhances its reproductive success relative to other possible traits (Futuyma, 1998). On the other hand, the process of adaptation refers to ongoing evolutionary change that is driven by selection in a given environmental context. Therefore, an adaptive trait is the result of the process of adaptation.

Strictly speaking, a trait can only be assumed to be adaptive when it first appears. As time goes on, the trait may persist for any of three reasons. First, the trait might still be adaptive for the original reason (selective pressure remains). Second, in a case where the selective pressure has relaxed, the trait may persist passively (no longer adaptive) if there is no selection against it. Third, since its original appearance, other features may have become linked to the original trait so that even if the original selective pressure is relaxed, that trait persists because other processes that experience selection pressure depend upon it (no longer adaptive for the original reason, i.e., an “exaptation”). Many evolutionary biologists do not accept the use of the term “adaptation” for a trait that falls into either of the latter two categories. Most scientists who specialize in evolution would evaluate the adaptive significance of a trait in both the context of its phylogenetic history and in the context of the environment in which the organism naturally lives (Gregory, 2008).

## The “just-so” problem

For the bulk of circadian-regulated events, we should consider whether a critical evolutionary biologist would agree that we circadian biologists have demonstrated that the accurate timing of clock-regulated events enhances fitness. Until the 1970s, it was common for biologists to interpret the phenomena they observed according to a line of reasoning that might be paraphrased, “if a biological phenomenon is present, it must have been selected by evolution and therefore of adaptive significance.” Even sophisticated scientists can fall prey to this type of reasoning (Gould and Lewontin, 1979). As further evidence of this misunderstanding, an integration of data from the Internet by the probabilistic artificial intelligence natural language tool ChatGPT in response to the query “What evidence is there that circadian clocks confer fitness?” conflates the existence of specific clock-controlled processes with the conclusion that circadian clocks confer fitness, even in the absence of direct evidence for clock-enhanced reproductive success (Supplemental Dataset S1).

A seminal paper by Stephen Jay Gould and Richard Lewontin (1979) entitled “The Spandrels of San Marco” labeled this line of thinking an “adaptationist program” that provided “Just-so” explanations of biological phenomena that are intellectually satisfying but might have little basis in the reality of the history of evolution. For example, many biological phenomena might have evolved (1) as a random trait that was neither adaptive nor non-adaptive, (2) as a trait physiologically linked to another trait where the linkage is either not currently present or not obvious, (3) as a trait that was once adaptive but is no longer for the original function (e.g., the tailbone and/or appendix of humans), (4) as a trait that evolved for one purpose but later was recruited to another task, and so on. To illustrate the concept, consider the case of human noses and spectacles—since noses provide such an excellent platform for mounting spectacles, it would be easy to assume (in the absence of knowledge of the history of noses and spectacles) that noses evolved so as to provide a place for spectacles to reside (Gould and Lewontin, 1979). Could the circadian system be another such case?

## Natural selection is acting on . . . what?

**Figure 1.**
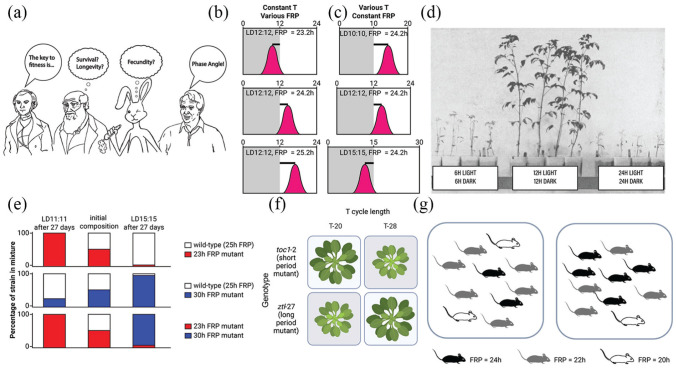
The key significance of phase angle and examples of competition assays. (a) For circadian clocks, phase angle (the phase relationship of the biological clock to the environmental cycle) is the key to fitness. (b) Phase angle is a function of the FRP of a circadian rhythm, here shown as the phase relationship of a rhythm to a consistent 12:12 light/dark cycle (LD12:12) as FRP is varied. (c) Phase angle of a circadian rhythm is a function of the period (T) of an environmental cycle, here shown as the phase relationship of a circadian rhythm with an intrinsic FRP = 24.2 h as it entrains to environmental cycles of different T (T-20 = LD10:10, T-24 = LD12:12, T-30 = LD15:15). (d) A classic test of the effect of T-cycles (T-12, T-24, and T-48) on the growth of tomato plants (Highkin and Hanson, 1954), reprinted by permission of *Plant Physiology*. (e) Competing cyanobacteria: when strains of cyanobacteria with different FRPs (FRPs of 23, 25, and 30 h) are competed under different T-cycles (T-22 and T-30), the strains whose FRPs “resonate” with the environmental cycles (adopt optimal phase angles) outcompete other strains. Mutant strains are able to outcompete wild-type strains if their FRPs harmonize more appropriately with the T-cycle than the FRP of the wild-type strain (Ouyang et al., 1998; Woelfle et al., 2004). See the “The Competition Assay applied to Cyanobacteria” section. (f) Competing plants: similar to the case of cyanobacteria, the growth and mortality of *Arabidopsis* plants are promoted under aligned combinations of FRP and T-cycles (Dodd et al., 2005). See “The Competition Assay Applied to *Arabidopsis*” section. (g) Competing mice: In a competition experiment, the *Csnk1e*^tau^ allele frequency—causing FRP to shorten by 2 h in heterozygote mice and by 4 h in homozygote mice—gradually decreases in mouse populations over multiple generations (Spoelstra et al., 2016). Figures depict proportions of the genotype in replicated populations in outdoor enclosures. See the “The competition assay applied to mice” section. Abbreviation: FRP = free-running period.

**Figure 2.**
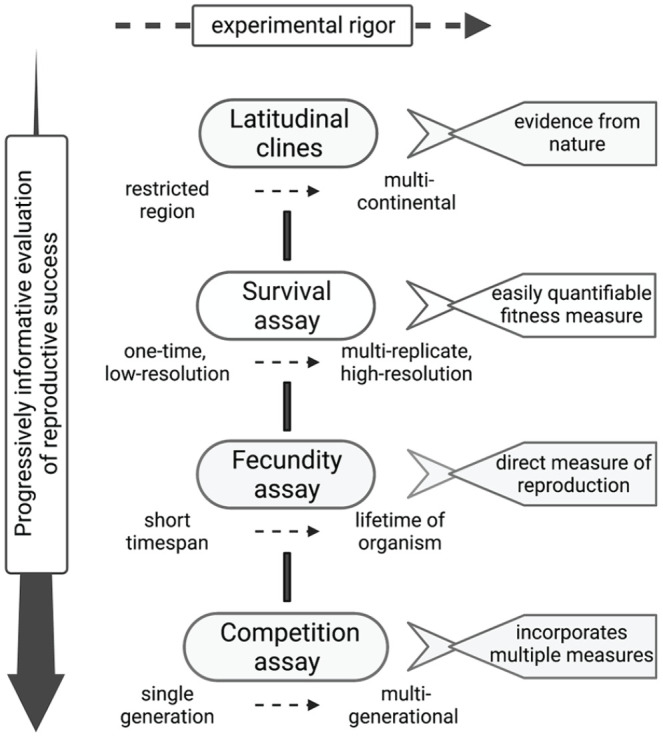
Supporting evidence for the adaptiveness of clocks. A schematic depicting various assays used for inferring the evolutionary fitness of clocks ordered in a hierarchy based on the information they provide on reproductive success (vertical) and variations in these assays based on experimental rigor involved (horizontal). More information on advantages and drawbacks is included in Table 1.

**Figure 3. fig3-07487304231219206:**
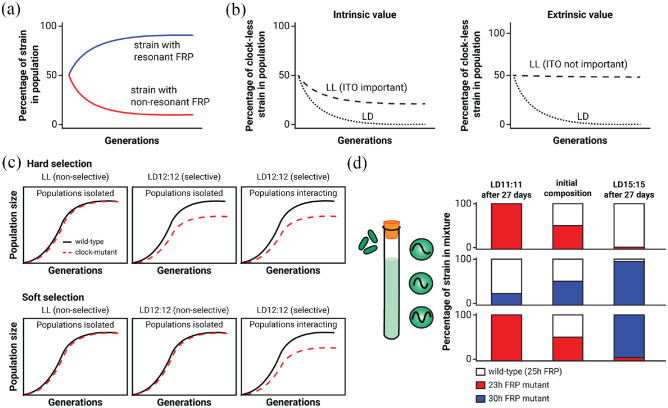
Competition assay applied to cyanobacteria. (a) Competing cyanobacteria: when strains of cyanobacteria with different FRPs are competed under different T-cycles, the strains whose FRPs “resonate” with the environmental cycles are predicted to outcompete the strains whose FRPs do not resonate (Ouyang et al., 1998; Woelfle et al., 2004; Ma et al., 2013). (b) Competing cyanobacteria: When a wild-type strain of cyanobacteria is competed against a clock-less mutant, the wild-type strain is predicted to win in T-24 LD conditions. In LL, wild type should win if maintenance of an ITO is important (intrinsic), or has no selective impact in LL if ITO is less important (extrinsic). (c) The winner of a competition experiment can be determined through hard selection (top panel) and/or soft selection (bottom panel) on the basis of comparing single-strain populations versus mixed-strain populations. In the case of hard selection, the environment (LD12:12) negatively impacts the fitness of a strain (clock-less), but not that of wild type, and this effect can be observed in single-strain, non-competing population. This results in lower growth of the clock-less strain under the selective LD12:12 condition but not in the non-selective LL and can eventually lead to wild type outcompeting the clock-less strain in a mixed culture. On the other hand, no difference in growth is observed between LD12:12 and LL in single-strain populations under soft selection. Only when the two strains are competed against each other in mixed-strain populations is the growth and fitness of the clock-less strain reduced. (d) Competing cyanobacteria: when strains of cyanobacteria with different FRPs (FRPs of 23, 25, and 30 h) are competed under different T-cycles (T-22 and T-30), whichever strain has the FRP that “resonates” the most with the environmental cycle (and likely adopts the optimal phase angle of entrainment) is the winner of the competition (Ouyang et al., 1998; Woelfle et al., 2004). Abbreviations: FRP = free-running period; ITO = internal temporal order.

**Figure 4. fig4-07487304231219206:**
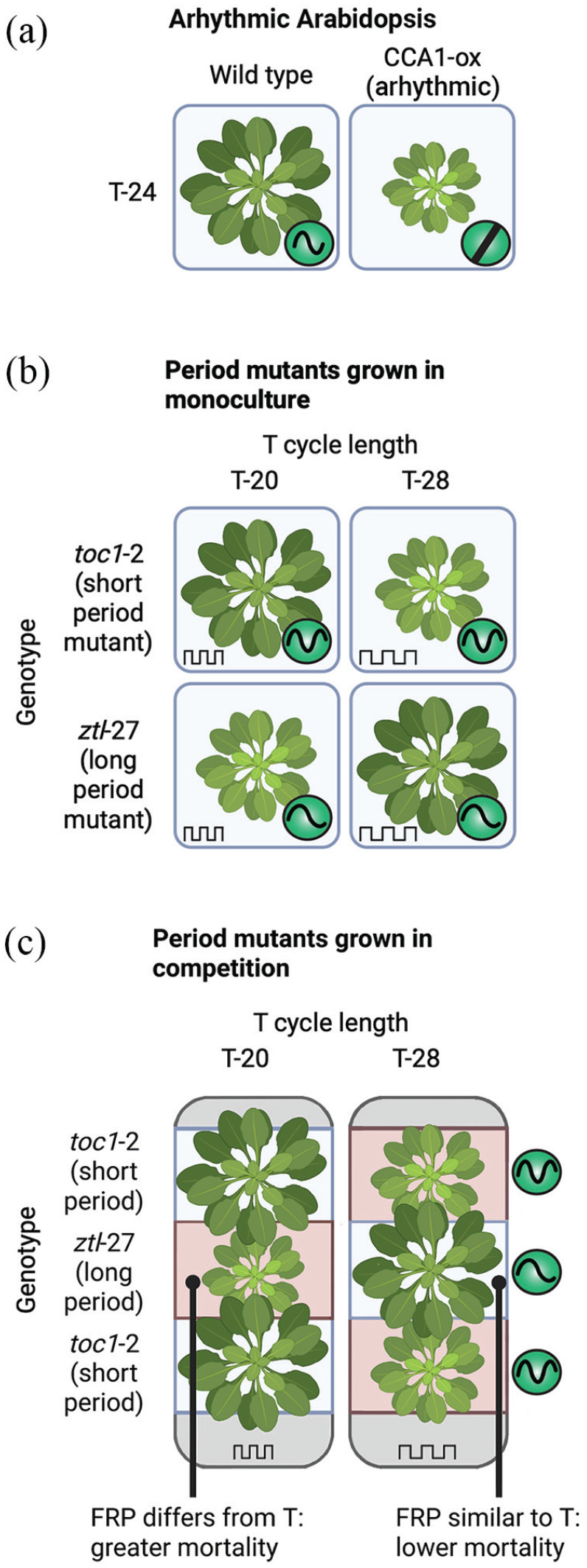
Circadian clocks contribute to the fitness of *Arabidopsis* plants. (a) Decreased growth and biomass accumulation of arhythmic (CCA1-ox) plants compared to the wild type, grown under T-24 (LD12:12). (b) For *Arabidopsis* plants grown in single-strain monocultures, there was decreased growth and biomass accumulation when the endogenous circadian period length differed from the environmental T-cycle length. (c) For *Arabidopsis* period-length mutants grown in competition, there was also decreased growth and biomass accumulation, combined with greater mortality, when the endogenous circadian period differed from the T-cycle length. Diagram shows representative plants. Abbreviation: FRP = free-running period.

As noted by many, but perhaps most clearly by Roenneberg and Merrow, “Evolution has shaped circadian clocks in a cyclic world; temporal constancy of environmental qualities must have been an extremely rare exception. It is therefore the mechanism of entrainment that has evolved and not sustained rhythmicity in constant conditions” (Roenneberg and Merrow, 2002). The original adaptation of circadian clocks was likely to enhance reproductive fitness by providing an internal estimate of external time so that biological events are phased to an optimal time of the 24-h day (Johnson et al., 2004). In other words, phase angle must have been the original property that was acted upon by natural selection (Figure 1a). Temperature compensation is a necessary part of the mechanism that synergistically ensures that phase angle is conserved (Johnson et al., 2004). Phase angle “conservation” is not necessarily rigid; plasticity of phase angle as a function of season or the daily fluctuating environment may be important for many organisms living in the real world (Pittendrigh, 1979). As we know from entrainment theory, phase angle can be altered by varying the intrinsic period of the biological clock (the free-running period [FRP]) and/or by varying the period of the environmental cycle (“T,” Figure 1b and 1c). Therefore, phase angle can be experimentally manipulated by altering either the FRP or, in the laboratory, the T. Of historical interest, one of the first studies known to us that used T-cycles to address questions of adaptive significance was a study of tomato growth, shown in Figure 1d (Highkin and Hanson, 1954). Remarkably, tomato plants cultivated on an LD12:12 cycle outgrew those on LD6:6 h or LD24:24, even though all these plants received equal amounts of light and darkness over time. Manipulations of FRP and T occur frequently in the tests of adaptive fitness that we describe in the following section (Figures 2 [Fig fig3-07487304231219206][Fig fig4-07487304231219206]-5), especially in competition assays (Figures 1e-1g and 3a).

**Table 1.** Pros and cons of assay types for inferring the evolutionary fitness of clocks.

Assay Type	Advantages	Drawbacks
Latitudinal clines	Evidence from natural environments	Correlational
Survival/Longevity	Intuitively, quantifying survival up to reproductive age is a good estimate of reproductive success	Time-consuming
		Not a direct measure of reproductive success
Fecundity	Directly linked to reproductive success	May not determine reproductive success in isolation in all scenarios
	Can provide a mechanistic basis for fitness phenotypes	Difficult to assay as it varies largely based on age, environment, etc.
Competition	Incorporates aspects of survival and fecundity	Output is in the context of competitive ability in an environment with limited resources
	With some experimental models, can be used to study change in allele frequency over multiple generations	Outcome can be influenced by interactions between competing organisms and hence may not reflect absolute fitness

**Figure 5.**
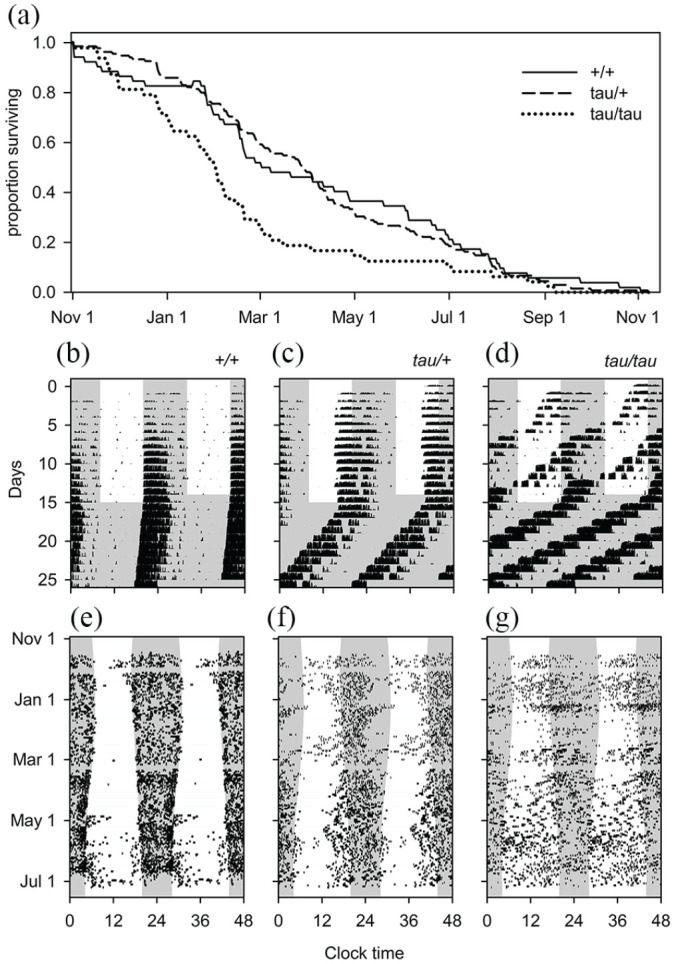
Circadian clocks contribute to the fitness of mice. (a) Survival curve of mice with mutant “tau” (*Csnk1e*^tau^) alleles in a competition experiment in outdoor enclosures. (a) The 3 lines show the surviving proportion of the 3 genotypes: wild-type (*+/+*) mice with a ~24-h FRP, heterozygote (*tau/+*) mice with a ~22-h FRP, and homozygote (*tau/tau*) mice with a ~20-h FRP. The latter genotype lives significantly shorter. (b-d) Laboratory (running wheel) activity patterns of the 3 mouse genotypes (which are highly similar to those of tau-mutant hamsters; Loudon et al., 2007) in LD12:12 conditions during day 1-15 and free-running in DD between day 15 and 25, clearly showing the effect of the mutation on the FRP. The misalignment of heterozygous mice to dissonant LD conditions strongly compromises longevity. (e-g) Outdoor enclosure activity patterns (transponder recording at feeding stations) of the three genotypes (tested over 424 days). Here, rhythms are less pronounced and in contrast to heterozygous mice in the laboratory, and longevity is compromised in homozygous mutant mice. This may be caused by other selective pressures, for example, by aerial predation. Abbreviation: FRP = free-running period.

We can refer to the optimization of phase angle as an adaptation to “extrinsic” conditions. However, some researchers have proposed that circadian clocks may additionally provide an “intrinsic” adaptive value (Pittendrigh, 1993; Paranjpe et al., 2003). That is, circadian pacemakers may have evolved to become an intrinsic part of internal temporal organization and consequently provide an “internal temporal order” (“ITO” in Figure 3b). For example, temporal programs could be valuable to separate mutually incompatible processes in time and/or to optimize the synthesis of complex products that might have toxic intermediates. As such, the intrinsic value may have become intertwined with other traits in addition to their original role for phase-angle adaptation to environmental cycles, all of which influence reproductive fitness. Note that many rigorous evolutionary biologists would no longer consider an intrinsic value for clocks to be an adaptation to the original pressure if their extrinsic value has been lost. However, if clocks retain extrinsic value and additionally accrue intrinsic value, then they would still be considered an adaptation to that original selective pressure.

An extrinsic value derived from an optimal phase angle achieved under entrained conditions is expected to disappear under constant environments where the selective pressure is absent (e.g. “LL, ITO not important” in Figure 3b). On the other hand, should circadian clocks acquire intrinsic value for internal temporal programming, they might continue to be of adaptive value to an organism in constant environments (e.g. “LL, ITO important” in Figure 3b). In support of an “intrinsic-value” hypothesis, populations of *Drosophila melanogaster* raised for hundreds of generations in constant conditions retain rhythmicity and the ability to entrain to various LD cycles, implying that even in the absence of environmental selection, the circadian system is beneficial (Paranjpe et al., 2003). However, it is possible that this experiment was not of sufficient duration to answer the question—a counterexample being cave animals that have lost robust behavioral rhythmicity in the constant environment of caverns (Blume et al., 1962; Beale et al., 2016; Abhilash et al., 2017).

## Criteria for establishing adaptive fitness

Multiple lines of evidence may be used to support the hypothesis that clocks enhance adaptive fitness, such as adaptations to latitudinal clines, performance in survival assays, fecundity assays, and competition assays. Each of these kinds of evidence has strengths and weaknesses, as described in this section and in Figure 2 and Table 1.

### Latitudinal Clines

One way to assess the ongoing adaptive value of circadian clocks would be to search for evidence of natural selection acting upon circadian parameters in nature. One type of evidence could be gradations in circadian rhythm properties where selective strength varies over a gradient of a relevant environmental condition. An excellent example of this type of environmental condition is the latitudinal changes in annual day length and temperature (Hut et al., 2013). Day length and temperature are both highly relevant to the daily clock and its associated property of photoperiodic time measurement. Moreover, the continued evolution of circadian clocks in the context of climate change is highly likely to occur differentially along latitudinal clines in organisms that cannot easily change their geographical range (Jabbur and Johnson, 2022).

In support of the prediction that these gradients influence clock properties, there is a positive correlation between the circadian period and the latitude from which samples of the plant *Arabidopsis* have been isolated from nature across a wide latitudinal range (16°N-66°N, Michael et al., 2003). Curiously, this correlation was not replicated in a study looking at a *narrower* range of latitudes (55°N-63°N, Rees et al., 2021), which instead found that *Arabidopsis* plants collected from southern regions had longer FRPs than those collected from northern regions, nor in a separate study (Edwards et al., 2005) that looked at a wide range of latitudes (15°N-59°N) but limited itself to 27 *Arabidopsis* accessions. Together, these 3 studies suggest that large sample sizes might be necessary to identify the presence of latitudinal clines in FRP, and that within narrow ranges of latitudes, perhaps other factors provide stronger selective forces, leading to positive or negative correlations of period and latitude depending on the range measured. Indeed, a recent study of plants within a 1º latitudinal range in the Rocky Mountains (USA) found that an *Arabidopsis* relative, *Boechera stricta*, had a strong correlation between FRP and elevation but not latitude (McMinn et al., 2022).

Besides *Arabidopsis*, positive correlations between period and latitude are also observed in wild plant populations of the annual (but not perennial) *Mimulus guttatus* and domesticated cultivars of soybean (Greenham et al., 2017), for which the FRP increased the further north the plants were collected. The case of soybean is particularly interesting, as the plant originated in China, and the cultivars used in the study were those from North America where it was introduced only recently (19th century). The differences in FRP thus likely arose from human selection to improve their performance for varying agricultural practices in differing latitudes and human cultures. The relationship between latitude and properties of the circadian clock also appears to be important for crop production in other plants besides soybean. For example, the domestication of tomato has involved its cultivation at more northerly latitudes than its ancestral wild species. This cultivation at more northerly latitudes is associated with selection during plant breeding for progressively longer circadian FRPs caused by mutations in circadian clock genes such as *EID1* and *LNK2* (Müller et al., 2016, 2018). This might be associated with acute sensitivity of tomato to the timing of light exposure (Highkin and Hanson, 1954). In other species, however, selection might lead to changes in clock outputs rather than in the clock itself: In the case of cucumbers, comparisons between wild Indian accessions, semiwild Chinese accessions, and domesticated Eurasian and East-Asian cultivars showed stark differences between the expression of the *FLOWERING TIME* (*FT*) gene, which arise from differences in regulatory elements that are upstream of the FT coding sequence. Accessions from high latitudes (Eurasian and East-Asian) have a shorter upstream region of FT, whereas those from low latitudes (Indian and Chinese) have a longer upstream region (Wang et al., 2020).

In animals, latitudinal clines of clock properties in drosophilids have been a frequent subject of interest. For example, among Japanese strains of *Drosophila auraria* (34.2°N-42.9°N), circadian properties (phase angle, FRP lability, and PRC amplitude) differed between the high- and low-latitude strains (Pittendrigh and Takamura, 1989). Also, the eclosion rhythms in *Drosophila littoralis* (30°N-70°N) and *D. subobscura* (56°N-63°N) were shown to have clinal variation in phase angle and FRP (Lankinen, 1986, 1993). Genetic studies in *D. melanogaster* uncovered latitudinal clines in polymorphisms of the *period* and *timeless* genes that have been extensively analyzed. For the *per* gene, there are differing lengths of a threonine-glycine (Thr-Gly) encoding repeat region of the *per* gene that vary over the latitudes of Europe (Costa et al., 1992). Statistical tests involving the patterns of nucleotide variation around this region in *D. melanogaster* revealed evidence for weak balancing selection (Rosato et al., 1997). Interestingly, the length variants in *per* show different circadian temperature compensation properties that appear to be adaptations to the geographical regions in which each type of Thr-Gly allele predominates. Thus, balancing selection at the nucleotide level appears to be mirrored at the behavioral level (Sawyer et al., 1997).

In the case of the *timeless* gene of *D. melanogaster*, there are two allelic forms, *ls-tim* and *s-tim*, that differ in protein length and light sensitivity (Sandrelli et al., 2007). In Europe, the frequency of *ls-tim* generally increases from north to south, and this would initially appear to represent a latitudinal cline. However, it is actually a distance cline with frequencies of *ls-tim* correlating with the overland distance from a point in south-eastern Italy where the frequency of *ls-tim* is at its highest (Tauber et al., 2007; Zonato et al., 2018). This suggests that the *ls-tim* mutation initially arose in that area, an example of a founder effect, and has spread relatively recently with estimates from phylogenies suggesting that the mutation is between 300 and 3000 years old (Tauber et al., 2007; Zonato et al., 2018). The *ls-tim* flies are less sensitive to light and significantly more rhythmic under continuous light but also show higher levels of diapause than *ls-tim* under all photoperiods than the ancestral *s-tim* variant (Sandrelli et al., 2007; Tauber et al., 2007; Kyriacou et al., 2008; Deppisch et al., 2022). The *ls-tim* phenotype of reduced circadian photosensitivity is more adaptive in northern Europe, especially during summers with extremely long photoperiods at higher latitudes that would be expected to make flies arhythmic (Sandrelli et al., 2007; Deppisch et al., 2022). The enhanced diapause of *ls-tim* also is more adaptive in the north during autumn/winter when colder temperatures arrive earlier than in southern Europe and when photoperiods are still relatively long (Tauber et al., 2007). Given that *ls-tim* likely arose in southern Europe, these light-sensitive phenotypes would be advantageous compared to the ancestral allele in any seasonal environment such as Europe and may explain the *ls-tim* spread from its geographic origin in the south. This scenario is also supported by genomic neutrality tests which reveal that the *ls-tim* allele, unlike the *per* Thr-Gly region, is under directional, not balancing selection (Tauber et al., 2007; Zonato et al., 2018).

While these observations of latitudinal variation in circadian clock properties are intriguing, disentangling the impact of different environmental and geographical factors that may be involved in creating such clines can be challenging. While geographical variation in clock gene allelic frequencies can be correlated with phenotypic traits that are likely to be relevant, such as circadian and seasonal timing, it is possible that there are other untested phenotypic and ecological factors that may also contribute to the polymorphisms (Hut et al., 2013). However, one useful feature of these studies is that applying neutrality tests to the genomic region of interest will give a good idea if that region is a focus for selection, thereby justifying further effort in trying to understand the selective forces. Without such a genomic signature, the study becomes merely another “Just-so” story.

#### Strengths

The major strength of this approach is that clinal variants are a product of selection in nature rather than under artificial lab conditions (Figure 2 and Table 1).

#### Weaknesses

Unfortunately, clinal studies are based on correlations. Causal variants are expected to correlate closely with environmental selection pressures and have a quantitative genetic basis, while neutral variants do not. Therefore, predictable variation in clock properties such as phase angle, FRP, and so on, can inform us of local adaptation to selection pressures upon clocks that arise from latitudinal photoperiod variation. However, distinguishing between causal variants and random variations along latitudinal vs distance clines can be challenging.

### Survival/Longevity and Growth/Developmental Rates

Intuitively, one might assume that if the biological clock helps to optimize an organism’s relationship with its environment, it should also optimize its survival/longevity. Enhanced longevity extends the presence of the gene set of an individual and the likelihood of producing more copies of it within a population. Nevertheless, while this measure might correlate with fitness in certain species (at least minimal survival is necessary for reproduction!), survival is not the same as reproductive success as exemplified by the analogy of Michael Menaker described in the first paragraph of the “Fitness and Adaptation” section. In a pioneering and heroically intensive study, Patricia DeCoursey investigated the predator-avoidance hypothesis experimentally in natural populations of diurnal chipmunks by tracking the survival in nature of chipmunks with lesioned suprachiasmatic nucleus (SCN-X) as compared with sham-operated and control chipmunks using radio telemetry (DeCoursey et al., 2000). Remarkably, her findings revealed that SCN-lesioned chipmunks exhibited reduced survival and higher rates of predation than the other groups. The compromised longevity observed in SCN-X chipmunks was likely attributed to their altered behavior during the night. The telemetric data indicated that despite staying inside their burrows during the night, SCN-X individuals displayed increased activity within the burrow, potentially betraying their presence to predatory weasels. DeCoursey concluded that an intact circadian system enhanced the survival of chipmunks, but reproductive success was not explicitly tested.

In insects, an early example of survival measures was that of Pittendrigh and Minis (1972), who tested the longevity of *Drosophila pseudoobscura* adults in constant light or T-cycles of 21, 24, and 27 h. They reported that flies lived significantly longer in T-24, implying an optimal “resonance” of the internal clock’s period with the period of the environmental cycle. However, a “fly in the ointment” was that the result was not replicable after Pittendrigh’s laboratory moved from Princeton University to Stanford University (Dr. Terry Page, personal communication). A few years later, Jürgen Aschoff’s laboratory reported a similar approach to test the longevity of blowflies, which died more rapidly on non-24-h light/dark cycles than on T-24 cycles (von Saint Paul and Aschoff, 1978). Another study using different combinations of photoperiods and T-cycles found that flesh flies (*Sarcophaga*) develop most rapidly under long photoperiods of a T-24 cycle (Saunders, 1972). Investigating the longevity of wild-type (WT) *D. melanogaster* from a large, outbred population as well as from an inbred WT Canton S population, Kumar and co-researchers reported that a sizable fraction (20%-25%) was arhythmic in activity-rest under DD conditions (Kumar et al., 2005). Flies from both populations that lacked clear rhythmicity had a significantly shorter lifespan than those that were rhythmic, suggesting that the absence of a circadian rhythm in DD negatively impacted physiological health in *D. melanogaster*. A critical argument against the conclusions of this study is that the arhythmic flies in both populations might have suboptimal health, and impaired rhythmicity could have been a simple consequence of poor health.

Not all published studies support the credo of clock-enhanced fitness. For example, comparisons of the lifespan of WT *D. melanogaster* flies with clock FRP-mutant flies (clock-null *per*^0^, short-period *per*^T^, and long-period *per*^L^) showed that while WT flies lived slightly longer under a T-24 cycle, there were no differences in lifespan among the strains under a T-16 cycle that would have been expected to resonate with the FRP of *per*^T^ (Klarsfeld and Rouyer, 1998). Similarly, in jewel wasps with naturally varying FRPs, discordance between the FRP and T-cycle length did not reduce longevity (Floessner et al., 2019). While earlier studies have suggested that differences in light regimes can modulate the trade-off between lifespan and fecundity (Sheeba et al., 2000), a recent study also showed that flies maintained in constant darkness lived longer than those in light-dark laboratory conditions, irrespective of changes in behavior such as feeding, activity, and fecundity (Johnson et al., 2023). Interestingly, blind flies did not live longer under constant darkness, suggesting the existence of a perceptual component affecting aging independent of rhythms. Our tentative conclusion from these various studies is that in insects, circadian clocks may only conditionally contribute to longevity differences.

As in the experiments testing the impact of T-cycles on blowflies, longevity in hamsters is affected if the FRP is not resonating with the T-cycle. However, “tau” mutant hamsters harboring the *Csnk1e*^tau^ allele, with endogenous FRPs of 22/20 h (heterozygous and homozygous, for the *Csnk1e*^tau^ mutation, respectively; Ralph and Menaker, 1988), only face compromised longevity when entrained to non-resonant T-cycles. In laboratory experiments, heterozygous (22-h FRP) mutants that entrained to a T-24 h cycle with an “incorrect” phase angle—a “permanent jet lag”—develop severe health issues (Martino et al., 2008). Homozygous mutant (20-h FRP) hamsters that did not entrain to T-24 but free-ran did not develop any health problems. Longevity is restored, and health problems are absent when heterozygous animals are kept in a T-22 cycle (Hurd and Ralph, 1998; Martino et al., 2008). This observation agrees with the reported compromised longevity in heterozygous and uncompromised longevity in homozygous mutants (Hurd and Ralph, 1998; Oklejewicz and Daan, 2002) and is consistent with the effects of natural variation of FRP in WT mice and primates on lifespan, where individuals that express FRPs closest to 24 h living longer (Libert et al., 2012; Hozer et al., 2020). Taken together, these observations support an interpretation that phase angle is the key property upon which natural selection acts (Figure 1a). However, such selective effects may only manifest in laboratory conditions, where benefits of entrainment to cycles different from endogenous periods may be mostly absent. Under natural conditions, where entrainment may rescue individuals from elevated day/night-dependent predation, effects on longevity for individuals with various FRPs may be very different.

In plants, longevity and the rate of development are often tightly linked. Several studies from the 1950s addressed the impact of optimal clock-environment resonance on growth. For example, tomatoes were found to grow optimally when maintained on light/dark cycles that were similar to those encountered in nature; in other words, tomatoes cultivated under LD12:12 light/dark cycles outgrew those on LD6:6 h or LD24:24 (Figure 1d; Highkin and Hanson, 1954). Remarkably, tomato plants cultivated on an LD12:12 cycle grew even faster than those under continuous light, even though the plants under constant light were receiving twice as much photonic energy (Hillman, 1956). Those data indicate that tomato plants are optimally adapted to growth in light/dark cycles with characteristics similar to those found in nature (i.e., a period of 24 h), implying that a circadian timekeeper is responsible for the adaptation. In the model plant *Arabidopsis*, experiments using manipulations of the T-cycle length and period mutants indicate that growth is generally greater when the T-cycle length is aligned with the circadian period (Dodd et al., 2005; Graf et al., 2010). For certain circadian clock mutants, this relationship does not occur (e.g., *toc1*-2 and *ztl*-3 mutants grow faster under T-24 than their cognate T-cycle lengths; Graf et al., 2010). These complexities might occur because certain *Arabidopsis* circadian clock components regulate large numbers of genes, presenting challenges for disentangling phenotypes caused by circadian regulation versus perturbed gene expression profiles. These issues underscore the importance of considering a variety of mutant alleles and circadian phenotypes in such experiments and moreover recognizing the possibility that phenotypes might depend on specific growing conditions.

#### Strengths

An advantage of using growth/developmental rate and longevity as indicators is that these measures are relatively easy to assess. A justification for using this convenient gauge of “fitness” is that the survival of an organism up to reproductive age is a fundamental requirement for successful reproduction (Figure 2 and Table 1).

#### Weaknesses

Growth and longevity are not direct measures of fitness, and therefore, results obtained from these types of studies may be tangential. In the case of plants, the extent to which altered T-cycles impact growth has been tested in only a limited range of plant species. Therefore, the extent to which the phenotypes reported by Highkin, Hanson, and Hillman are generalizable among plant species is uncertain. Similarly in insects, depending on the particular species examined, there can be a trade-off between development time and survival, and thus, these measures may not indicate an overall impact on fitness.

### Fecundity

A measure of reproductive success that is more direct than survival/growth is to quantify fecundity (Table 1). There are various approaches to the quantification of fecundity, including measurement of viable offspring numbers, measurement of production of viable gametes or zygotes, and/or investigation of mating success (Ågrena et al., 2013; Alif et al., 2022). A related but less direct measure of fecundity is that of insemination frequency, which can provide an underlying explanation for altered reproductive success (Anderson, 1974).

Investigations of the impact of circadian clock function upon reproductive fitness have had mixed success, particularly in *D. melanogaster*. For example, a study investigating the reproduction of clock mutants (*per*, *tim*, *Clk*, and *cyc*) found that disrupted circadian rhythmicity is associated with male fruit flies having decreased sperm production (Beaver et al., 2002). Furthermore, *per* and *tim* mutations in female fruit flies affected the production of mature oocytes. Comparing the fecundity of flies under LD (behaviorally rhythmic) and LL (behaviorally arhythmic, but high expression of clock proteins) conditions yielded comparable numbers of mature oocytes, demonstrating that the level of expression of *per* and *tim* in the ovary—rather than their oscillation—is important for fecundity (Beaver et al., 2003). Similarly, *Bmal1*^−/−^ and *mPer* mutant mice have health and fertility problems that are almost certainly not clock-specific effects, but more likely due to BMAL1 being a globally active transcription factor that determines myriad gene expression programs (Kondratov et al., 2006; Jiang et al., 2022). A synergistic conclusion was reached with a mating assay from a recent study that compared the mating frequencies of mosquitoes under two different light conditions: constant light versus a T-24 light-dark cycle. This study reported that mosquitoes exposed to constant light had a significantly lower mating frequency than those entrained to light-dark cycles (Wang et al., 2021), implying a role for entrainment of an underlying clock in the adaptive fitness of mosquitoes.

In plants, the quantity, viability, and germination characteristics of seeds have provided information about the role of the clock in fecundity. One study compared the quantity and germination rate of *Arabidopsis* seeds between WT and an arhythmic strain that overexpresses *CIRCADIAN CLOCK ASSOCIATED 1* (*CCA1*). The authors found that seed production and viability were reduced under a very short photoperiod (LD4:20, Green et al., 2002). However, such a photoperiod is unlikely to be encountered by flowering *Arabidopsis* in nature, and more ecologically relevant photoperiods (LD8:16 and LD16:8) did not alter seed production (Green et al., 2002). A separate study identified that several circadian clock components affect seed dormancy, which is an important seasonal regulator of germination (Penfield and Hall, 2009). Neither of these studies demonstrate that circadian rhythmicity or alignment of the phase of biological processes with the environment determines these phenotypes, and—as with mice and flies—the plants might be less healthy because circadian clock transcription factors in *Arabidopsis* are involved in the expression of a large number of genes (Nagel et al., 2015; Adams et al., 2018). The circadian clock also contributes to seed production by influencing pollination. For example, in sunflowers, the orientation of the capitulum (flowering head) is adjusted to track the sun during the day, with the circadian oscillator contributing to this process. Appropriate orientation of the capitulum raises its temperature, which is thought to encourage pollinator visitation (Atamian et al., 2016), thereby increasing seed set and therefore potential reproductive success (Creux et al., 2021). Disruption of circadian rhythms in *Nicotinana attenuata* has also been reported to reduce fitness by interfering with pollination success, perhaps through alteration of the angle of the flower, nectar production, or scent production (Yon et al., 2015, 2016).

#### Strengths

Studies of fecundity conducted with circadian clock mutants or under altered T-cycles have the potential to identify mechanisms that underlie alterations in reproductivity. Investigation of effects of circadian regulation upon fecundity could be informative in the context of climate change because reproductive processes of plants, including seasonal control of reproduction, are altered by environmental conditions that simulate future climates (O’Neill et al., 2019).

#### Weaknesses

The fecundity experiments reported earlier do not directly measure changes in circadian-gene allele frequency across generations, which would provide stronger evidence for fitness (Figure 2 and Table 1). Furthermore, interpretation of results can be complicated by circadian clock components functioning as transcriptional regulators that regulate myriad genes, but where the rhythmicity *per se* of the genes’ expression is not crucial for fecundity.

### The “Competition Assay” as a Rigorous Test of Adaptive Fitness

A defect of the pre-1990 experiments (longevity, survival, one-generation measures of fecundity, latitudinal clines) is that they were not direct measures of reproductive fitness/success. In the field of population biology, a “gold standard” assay for fitness is to compete individuals with differing characteristics against each other under natural or semi-natural conditions to determine which characteristics allow some individuals to outcompete other individuals in those conditions (Figures 1e, 1f, 1g, and 3a). The competing groups might be different species or different strains of the same species, and the assay is preferably conducted over multiple generations so that selection for characteristics is coupled to reproductivity (Table 1). For example, classical experiments by G. F. Gause in the 1930s introduced the competition assay between different microbial species, in his case, competitions between two different species of *Paramecia* or between two different species of yeast (Gause, 1934). Those studies established the Principle of Competitive Exclusion, which states that when different species compete for the same ecological niche, one species survives while the other expires under a given set of environmental conditions (Gause, 1934). In the case of tests of adaptive fitness for clocks described in the following section, the competitors in this assay have been different strains of the same species whose genetics confer different circadian properties (different FRPs in the examples provided in the following sections).

The Johnson laboratory pioneered the competition assay as a rigorous test of the fitness value conferred by a circadian clock with an appropriately resonant FRP. Inspired by the work of Gause, Levin, and Lenski, who used microorganisms in competition to address population biology questions (Gause, 1934; Levin, 1972; Lenski and Travisano, 1994), Johnson and coworkers enlisted cyanobacteria for these competition tests (Ouyang et al., 1998; Woelfle et al., 2004). For asexual microbes such as cyanobacteria, differential growth of one strain under competition with other strains over multiple generations is a good measure of reproductive fitness (Lenski and Travisano, 1994). Subsequent to these studies in cyanobacteria, Dodd et al. (2005) and Spoelstra et al. (2016) extended the concept of the competition assay as an assessment of the fitness advantage of clocks to plants (*Arabidopsis*) and mammals (mice) (Spoelstra et al. 2016, sections “The competition assay applied to *Arabidopsis*” and “The competition assay applied to mice”). All three of these competition studies took advantage of genetic mutations in clock genes that altered FRPs, and in addition, the cyanobacterial and *Arabidopsis* studies modified the periodicity of the laboratory environment with T-cycles. As described in the following section, all three studies reported that alleles of clock genes which confer non-resonating combinations of FRP with the environmental period—and therefore non-optimal phase angle—decreased the fitness (Ouyang et al., 1998; Woelfle et al., 2004; Dodd et al., 2005; Spoelstra et al., 2016).

In the application of competition assays, a cautionary note is that a phenotype can disappear from a mixed population simply because of random genetic drift, that is, chance events that can lead to differential reproductive success and a consequent loss of variation. For phenotypes based on genetic differences, in any population that is given enough time, one allele will drift toward fixation regardless of whether there is selection for it or not. In the case of no selection and merely random drift, the probability that an allele will be fixed in a population is equal to its starting frequency (Futuyma and Kirkpatrick, 2009). In the reported experiments with cyanobacteria, initial proportions of the two competing strains were set to approximate 50% for each of the two strains. The “bottom line” is that in the examples described in the following sections, there is a chance that the “winning” populations could be false positives. The key to avoiding the random drift problem lies in conducting the experiments with multiple replicates of sufficiently large populations so as to confirm that the winners in the competition are outcompeting the losers due to a selective advantage and not due to genetic drift.

#### The Competition Assay Applied to Cyanobacteria

The first application of the competition assay to test the adaptive value of circadian programs compared various strains of the cyanobacterium *Synechococcus elongatus* that had different circadian properties, for example, different FRPs (Ouyang et al., 1998; Woelfle et al., 2004). In pure culture, the tested strains grew at about the same rate in constant light and in LD cycles, so there did not appear to be a significant advantage or disadvantage in having different circadian periods when the strains were grown individually. The fitness test was to mix different strains together and to grow them in competition to determine whether the composition of the population changed as a function of time. The cultures were diluted at intervals to allow growth to continue. Cyanobacteria are asexual, and therefore, differential growth of one strain under competition with other strains over multiple generations is a simple measure of both growth and reproductive success (Lenski and Travisano, 1994).

Different period mutant strains were used to answer the question, “Does having a period that is similar to the period of the environmental cycle enhance fitness?” The circadian phenotypes of the mutant strains used had FRPs of about ~22 h and ~30 h (Ouyang et al., 1998; Woelfle et al., 2004). The circadian phenotypes of these strains were caused by point mutations in three different clock genes, *kaiA*, *kaiB*, and *kaiC* (Ishiura et al., 1998; Woelfle et al., 2004). The WT has a period of about 25 h under these conditions. When each of the strains was mixed with another strain and grown together in competition, a pattern emerged that depended on the frequency of the LD cycle and the FRP. When grown on a T-22 cycle (LD11:11), the 22-h-period mutants could overtake either WT or the 30-h-period mutants in the mixed cultures (Figure 3a and 3d). On a T-30 cycle (LD15:15), the 30-h-period mutants could defeat either WT or the 22-h-period mutants. On an equinox T-24 cycle (LD12:12), the WT strain could outgrow either mutant (Ouyang et al., 1998). Note that over many cycles, each of these LD conditions have equal amounts of light and dark (which is important, as photosynthetic cyanobacteria derive energy from light); it is only the frequency of light versus dark that differs among the LD cycles. Clearly, the strain whose FRP optimally resonated with that of the LD cycle outcompeted the other strain (Figure 3a and 3d). Under a non-selective condition (in this case, constant light), each strain was able to maintain itself in the mixed cultures (Woelfle et al., 2004). Because the mutant strains could defeat the WT strain in LD cycles in which the environmental period is similar to the endogenous period, the differential effects that were observed are likely to result from the differences in the circadian clock and its entraining “resonance” to the environmental cycles and not to a nonspecific defect conferred by mutation. Because the growth rate of the various cyanobacterial strains in pure culture was not detectably different, these results are most likely an example of “soft selection” where the reduced fitness of one genotype occurs only under competition (Figure 3c, Futuyma, 1998; Reznick, 2016).

In a test of the extrinsic versus intrinsic value of the clock system of cyanobacteria, WT was pitted against an arhythmic strain (Woelfle et al., 2004). The arhythmic strain was defeated rapidly by WT in LD12:12, but under competition in constant light, the arhythmic strain grew slightly better than WT (Woelfle et al., 2004), which provided an example of the “ITO not important” case in Figure 3b. Taken together, the results from cyanobacteria support the conclusion that an intact clock system whose free-running period optimally harmonizes (optimal phase angle) with the environment significantly enhances the reproductive fitness of cyanobacteria in rhythmic environments. However, this same clock system provides no adaptive advantage in constant environments and may even be slightly detrimental to this organism. Therefore, the clock system does not appear to confer an intrinsic value (Figure 3b) for cyanobacteria in constant conditions.

Strengths of the competition studies in cyanobacteria are (1) they are a direct test of reproductive success, (2) clock mutants were able to outcompete WT under the environmental T-cycles that were optimal for the mutant FRPs (therefore, clock-specificity), and (3) the populations were large, which argues against the results being due to genetic drift.

Limitations of the cyanobacteria competition results are that (1) the experiments were conducted under non-natural laboratory conditions that might be too controlled to justifiably extend to a real-world environment, and (2) experiments in an asexual microbe may not be extrapolatable to sexual organisms (Figure 2 and Table 1).

#### The Competition Assay Applied to *Arabidopsis*

Circadian regulation appears to be ubiquitous among photosynthetic eukaryotes, including angiosperms, gymnosperms, bryophytes, and several algal species, implying that circadian regulation could be valuable to organisms whose energy acquisition is so directly linked to the daily cycle of sunlight and darkness. There is also conservation of specific molecular components of the circadian clock across these phylogenetic groups. The contribution of circadian regulation to the fitness of plants has been investigated using the plant model *Arabidopsis thaliana*. The experiments involved the cultivation of WT, FRP mutants, and arhythmic *Arabidopsis* lines under T-cycles while comparing physiological characteristics and survival. Both single strains grown alone (monocultures) and the competition between the strains with different FRPs have been tested (Dodd et al., 2005).

For example, Dodd and coworkers compared growth rates and biomass accumulation in monocultures of the WT and an overexpressor of the clock gene *CCA1* (*CCA1-*ox). *CCA1-*ox causes arhythmia of both the circadian oscillator and most clock-controlled processes. Under LD12:12 cycles, *CCA1*-ox accumulated lower biomass than the WT and grew more slowly (Figure 4a) (Dodd et al., 2005). In addition, circadian period length mutants of *Arabidopsis* (short-period *toc1* and long-period *ztl* mutant alleles) were grown under T-cycles that had a duration similar to or different from that of the endogenous FRP. Under these conditions, biomass accumulation was greater when the T-cycle duration was similar to the FRP and lower when the T-cycle length diverged from the FRP (Figure 4b). The circadian clock did not have a consistent effect upon the mortality of these single strains when cultivated alone. Dodd and coworkers also conducted competition experiments where short- (*toc1*-2) and long-period (*ztl*-27) mutant plants were grown in arrays of adjacent plants of the 2 genotypes under 2 different T-cycle lengths. In this experiment, mortality was consistently lower for mutants expressing an FRP that was similar to the T-cycle length and greater for mutants whose FRP differed from the T-cycle length (Figure 4c) (Dodd et al., 2005).

These data indicated that fitness is greater when the period of the circadian clock is aligned with the environmental T-cycle and competition is present. Misalignment between the FRP and T-cycle period reduces the likelihood of reaching the point of reproduction because mortality was altered. As with cyanobacteria, this suggests that resonance between the circadian clock and the environment contributes to organismal fitness. Because circadian regulation did not consistently affect mortality of plants grown in monocultures but consistently affected mortality when in competition, the *Arabidopsis* results appear to be primarily due to “soft selection” (Figure 3c).

Strengths of these experiments with *Arabidopsis* are that they (1) provided a direct test of survival while under competition, (2) allowed investigation of both “hard” and “soft” selection, and (3) provided information about essential aspects of physiology that contribute components of fitness.

Limitations are that the study (1) was not multigenerational, so it could not detect changes in gene frequency over multiple generations; (2) used an inbreeding plant species, preventing investigation of genetic segregation upon selection; (3) focused upon vegetative growth of a species that has distinct developmental stages (seed germination, vegetative growth, reproductive phase); and (4) occurred under controlled laboratory conditions so lacked the ability to extrapolate to naturally fluctuating environments (Figure 2 and Table 1).

#### The Competition Assay Applied to Insects

There have not been many examples of a competition assay being performed with insects to assess fitness benefits of clocks. A systematic study was conducted only recently with WT, clock mutant, and clock period mutant *D. melanogaster* spanning more than 50 generations and 2 years, measuring various fitness-related traits such as fertility, mating success, pre-adult survival, and reproductive output under both laboratory and semi-natural conditions (Horn et al., 2019). These results showed that in laboratory competition experiments, WT flies had a significant advantage over clock-null *per*^0^ flies, even in constant light conditions where both were behaviorally arhythmic. On the other hand, the study only partly confirmed a “circadian resonance” hypothesis, as only long-period mutants performed better under the longer T-cycle than under the 24-h cycle, whereas short-period mutants and clock mutants performed equally poorly under the shorter T-cycle and constant light as they did under the T-24 cycle. In addition, Horn and coworkers studied these fitness-related traits in fly populations in semi-natural (outdoor) environments (Horn et al., 2019). Under these conditions (T-24), WT flies outcompeted *per*^0^ flies as predicted. Nevertheless, the poor performance of the short-period mutants under shorter T-cycles under laboratory conditions clearly shows that additional factors—possibly unrelated to the clock—contribute to the fitness of the mutants (e.g., non-isogenized genetic backgrounds might have influenced the results, as no interbreeding with WT flies was carried out prior to experiments).

#### The Competition Assay Applied to Mice

Spoelstra and coworkers endeavored to extend the competition assay to mammals in semi-natural conditions. Rigorous competition experiments with mice in a more natural environment became possible with the development of circadian-clock mutant mice and the ability to keep track of them. Tracking mice in a semi-natural environment over a long time period was done with the use of subcutaneous passive micro transponders that send a unique code when activated by antenna coils, for example, near feeding stations. However, this technique can only be applied if mice are kept in enclosures, and hence, predation by terrestrial predators that were excluded by the walls of the enclosures was not possible. Nevertheless, mice under these open-air enclosure conditions still had to compete for food and reproduction, and they were exposed to aerial predation. To keep track of allele frequency changes in populations, newly generated mice were trapped, genotyped, and fitted with a transponder at regular intervals. The first rigorous experiment using this approach was done with mice with a less robust circadian rhythm conferred by the *mPer2*^Brdm1^ mutation (Daan et al., 2011). This *mPer2*^Brdm1^ mutant allele causes mice to have a relatively short circadian period in constant darkness, but more importantly, it weakens circadian rhythmicity. Contrary to the hypothesis that the *mPer2* mutant allele would be selected against and gradually disappear, the mutant allele fluctuated over time among the populations in the different enclosures, and its frequency ended at about the same value (0.54) as at the start of the experiment (Daan et al., 2011). Interestingly, there was a sex difference: Selection forces against the *mPer2* mutant allele were stronger in male mice.

The impact of a dissonant rhythm on Darwinian fitness was tested by Spoelstra et al. (2016) in a comparable setup with mice with the “*tau*” mutation (the R178 C mutation of Casein Kinase 1e, aka *Csnk1e*^tau^) competing against WT mice (Figure 5). Mice carrying this mutation have a shorter endogenous circadian period, caused by a gain of function in phosphorylation of circadian PER1 and PER2 proteins (Gallego et al., 2006) during a specific phase of the circadian cycle (Dey et al., 2005). Heterozygous mice express approximately a 22-h FRP when deprived of time-of-day information, while homozygous mice express a 20-h FRP. In small replicate populations, overall activity was fairly comparable between genotypes, with the mutant *Csnk1e*^tau^ allele causing mice to be more active in daytime. Over the course of 14 months, the mutant *Csnk1e*^tau^ allele frequency fell gradually from 0.5 to 0.2 in all populations. In the release cohort, for which the time of birth was known, homozygous mutant mice survived a significantly shorter time than heterozygous and WT mice (Figure 5a).

The effects of the *Csnk1e*^tau^ mutation on the FRP of mice are very similar with those in hamsters (Loudon et al., 2007; Figure 5b-5d). However, the shorter lifespan of homozygous mutant mice in the aforementioned experiment contrasts with compromised longevity of hamsters in lab experiments under T-24 cycles, in which heterozygous mutants live shorter (Hurd and Ralph, 1998; Oklejewicz and Daan, 2002; see also the “Survival/Longevity and Growth/Developmental Rates” section). Why in the outdoor enclosures do homozygous “tau” mice but not heterozygous mice live shorter lifetimes? This might be because the locomotor activity of all three genotypes in the outdoor-enclosure mouse populations was clearly modulated over 24 h by the natural light-dark cycle, forcing homozygous mice to delay their rhythm by 4 h every cycle, and perhaps this effect compromised their longevity. However, heterozygous mice did not show intermediate values for lifespan (Figure 5a and 5e-5g). This may indicate that other factors causing early death may select against homozygous mutant mice. An obvious control experiment testing the “permanent jet lag” hypothesis would be to study longevity in WT mice using shorter and longer T-cycles. This would solve the issue of pleiotropic effects, but unfortunately this experiment cannot be done in a natural setting where the day is obligated to occur on T-24!

In summary, compared to cyanobacteria, plants, and insects, these selection experiments in natural settings support the importance of resonant circadian rhythms for Darwinian fitness in mice. The major strength of these multigenerational competition experiments with mice in enclosures is that they were a direct test of reproductive success under semi-natural conditions in a sexually reproductive species that cannot self-fertilize.

Limitations are that there might have been confounding pleiotropic effects. For example, because casein kinase has multiple phosphorylation targets beyond clock proteins, the mutant *Csnk1e*^tau^ alleles may have physiological effects other than changing the FRP, and thereby affect mortality under the semi-natural conditions (even if these do not manifest under laboratory conditions). Note that modulating the T-cycle period (as was possible in the cyanobacteria and *Arabidopsis* competition experiments) is impossible in the outdoor enclosure experiments because the daily periodicity of nature cannot be changed (Figure 2 and Table 1).

## Discussion

### Criteria for Establishing Clock Fitness Advantage

As always, multiple approaches and criteria are better than a single rigid standard to establish a biological phenomenon. However, in the case of clock fitness, these approaches/criteria must focus upon reproductive success and not ancillary phenomena. We should avoid succumbing to a “Just-so” explanation epitomized by the ChatGPT response to the question of evidence for clock fitness (Supplemental Dataset S1). If possible with the experimental system, a multigenerational competition assay is preferable as a definitive gauge of reproductive success (Figure 2 and Table 1). When practical, manipulation of FRPs and T-cycles to correlate circadian resonance with optimal competitiveness regardless of genotype for both WT and mutants is most conclusive, and this has been achieved with both cyanobacteria and *Arabidopsis* (Ouyang et al., 1998; Woelfle et al., 2004; Dodd et al., 2005). Obviously, the ability to genetically manipulate the test organism is a boon and has been valuable in all the competition experiments discussed herein. Nonetheless, non-model organisms may have particular characteristics and/or economic importance that trump genetic manipulability in the choice of experimental subject.

### Selective Pressures That Led to the Evolution of Self-Sustained Circadian Rhythms: “Carrots and Sticks?”

How did circadian clocks evolve? What were the key selective pressures? We have cautioned herein against the danger of “Just-so” thinking, but we have to admit that speculations on the historical evolution of biological clocks are essentially “Just-so” stories. Therefore, with the caveat that we have no objective method to answer the question of how circadian clocks evolved historically on Earth, allow us to indulge in some tantalizing speculations (Johnson and Kyriacou, 2005). Fundamentally, we can imagine that timekeeper-promoting selective pressures may fall into two types: the “carrots” and the “sticks,” i.e., positive and negative pressures.

The carrots might be positive pressures to allow the anticipation of rhythmically available food sources or to synergize an internal temporal order (ITO in Figure 3). For example, plants depend upon photosynthesis which, in turn, depends upon rhythmically available sunlight, and perhaps optimal competitiveness for a plant entails getting the photosynthetic apparatus ready just in time for dawn. Honeybee “zeitgedachtnis” could be another example of the advantages of having an anticipatory timekeeper to remind a bee when a flower will be opening and ready for visiting. Regarding ITO, the example of “temporal separation” of photosynthesis and nitrogen fixation in cyanobacteria is relevant. In nitrogen-fixing unicellular bacteria, nitrogen fixation is often phased to occur at night. Nitrogen fixation is inhibited by low levels of oxygen, which poses a dilemma for unicellular photosynthetic bacteria because photosynthesis generates oxygen. Mitsui and coworkers proposed that the nocturnal phasing of N_2_ fixation was an adaptation to permit N_2_ fixation to occur when photosynthesis was not evolving oxygen (Mitsui et al., 1986). Because this time-dependent switching between photosynthesis and N_2_ fixation is beneficial in LD and LL, temporal separation in nitrogen-fixing cyanobacteria is therefore a potential example of the positive advantages of an ITO (but its fitness advantage has not yet been rigorously tested).

On the other hand, sticks might be conceived as negative pressures that punish organisms that are unable to keep track of time. For example, the “Escape from Light” hypothesis proposed by Colin Pittendrigh in 1965 falls into this category (Pittendrigh, 1965, 1993). Pittendrigh proposed that circadian clocks evolved as a result of a selective pressure generated by daily cycles of light and darkness in which the light was deleterious to the optimal growth of the organism. The hypothesis postulated that light (both ultraviolet and visible light spectra) can have deleterious effects on the genetics and biochemistry of cells and that organisms might respond to this photon bombardment by evolving (1) screening pigments, (2) colorless cellular components, and—most pertinently to this thesis—(3) a timing system to temporally segregate light-sensitive reactions to the nighttime when they will not be inhibited. An interesting extension of this line of thinking is to ask whether light is a carrot or a stick for photosynthetic organisms? The very first organisms to have evolved were probably not photosynthetic (Weiss et al., 2016), and therefore, their relationship with light was totally negative (i.e., an “Escape from Light” Stick?). With the evolution of photosynthesis (and cyanobacteria go back to the advent of life on Earth), however, the relationship with light became the source of energy and life (carrot) and a cause of deleterious mutations (stick) for photosynthetic organisms. Both carrot and stick became relevant!

### New Directions: Experimental Evolution of Rhythmicity From Arhythmic Organisms to Identify Effective Selective Pressures

Experimental evolution has been a successful approach in studying how traits can evolve and identifying new phenotypes and correlations between traits (Gibbs, 1999). Therefore, identifying selective pressures/conditions by which biological timekeepers can be experimentally evolved *de novo* in organisms that lack circadian clocks could provide valuable insights. An approach of this kind has not yet been reported, probably because the time required to experimentally evolve a process as complicated as a 24-h timekeeper could be prohibitive, especially since the probability of success is uncertain. Those considerations have discouraged the experimental evolution approach to circadian clock evolution. To date, the only related studies have been of selective pressures to modulate the clock properties of organisms that already have clocks, rather than deriving *de novo* timing systems. These include selection on divergent phasing of behavior (Pittendrigh, 1967; Kumar et al., 2007), stabilizing selection on the accuracy of phase angle (Kannan et al., 2012), long-term maintenance under constant environment conditions (e.g., constant light or darkness, consistent photoperiod [LD] cycles, and so on), or long-term exposure to semi-natural conditions (Sheeba et al., 1999; Imafuku and Haramura, 2011; Shindey et al., 2016; Dani and Sheeba, 2022). While these studies cannot be expected to provide the same depth of insight as the experimental evolution of circadian systems from arhythmic states, they have provided valuable insights into correlated evolution of clock properties (Dani et al., 2023).

### If Clocks Can Evolve, Can They DE-Evolve?

Related to the topic of experimental evolution under selective conditions (i.e., 24-h cycles), consider the case of organisms experimentally evolved under constant environments—do rhythms persist under non-selective environments devoid of time cues? Studies of *D. melanogaster* populations maintained for over 600 generations under LL conditions (where the flies are behaviorally arhythmic) nevertheless showed that the flies retain functional clocks, as evidenced by transferring the “LL-flies” to DD or LD. In DD, these LL flies exhibit robust free-running rhythms, and in LD, these LL flies functionally entrain to LD (Sheeba et al., 1999). Conversely, long-term studies of flies kept under DD have yielded unexpected results, with 2 different long-term studies from India and Japan (~330 generations and ~1340 generations, respectively) showing that fly populations evolved to have more robust rhythms after long exposures to DD (Shindey et al., 2016; Imafuku and Haramura, 2011). Therefore, under neither of these non-selective environmental conditions (long exposures to LL or DD) do clock properties “de-evolve.”

Perhaps the constant environment experiments in *Drosophila* simply have not been followed for a sufficient number of generations? Nature has already provided corollaries to these laboratory-based studies. As an example, Blind Mexican cavefish (*Astyanax mexicanus*) are a species of freshwater fish that have adapted to life in complete darkness in the caves of northeastern Mexico. These cavefish populations have disrupted biological clocks compared to their relatives dwelling in surface waters exposed to natural sunlight cycles. In addition, this disruption of clocks appears to have occurred via different molecular mechanisms in different populations (Beale et al., 2013; Mack et al., 2021).

### Is the Clock Still Evolving?

As with any evolutionary adaptation, the clock has been—and continues to be—subject to changing selective pressures and therefore is progressively transforming. One such property of the clock is its FRP, which long ago was likely to be much faster than 24 h because the earth is thought to have been spinning much faster in the distant past; for example, in the Cambrian, the daily cycle was likely to be ~21 h, but in the much more distant past, the day/night cycle might have been as short as 6-12 h (Spalding and Fischer, 2019). Cyanobacteria had already appeared at that geological time, and it is reasonable to assume that they had evolved in an endogenous period in those ancient times to adapt to the short T-cycle of day/night. Then as the eons rolled on and the earth’s rotation slowed, this provided a pressure that selected for an increasingly longer circadian period. Because this happened gradually over millions of years, there was plenty of time for the cyanobacterial clockwork to evolve to a more leisurely pace of ~24 h to adapt to the slowing earth.

Of more urgency, we have argued elsewhere that the cataclysmic (from evolutionary and geological perspectives) climate changes that we are currently undergoing will have a major impact upon photoperiodic time measurement and its partner, the circadian clock (Jabbur and Johnson, 2022). As temperatures become both higher and more erratic, organisms are confronted with a temporal mismatch between seasonal changes in average temperature and its previously reliable cue, photoperiod. This mismatch will occur because warm temperatures will occur during shorter photoperiods than before. In response to global warming, organisms who can change their geographical ranges may do so. Many species will not be able to rapidly adapt by moving to a new range, and they will be forced to change the time of year that they flower, mate, and so on or face extinction. Due to these rapid changes in climate, our biological timekeepers must adapt to allow appropriate responses to this meteorological metamorphosis. Studies of latitudinal clines and of domestication suggest that photoperiodic time measurement systems may be able to adapt by changing the FRP of the underlying clockwork and/or the critical photoperiod that induces migration, flowering, and so on. However, it is unclear whether organisms will have enough time to adapt by fundamental changes to their timekeeping processes before extinction, as the current rate of climate change is unprecedented. Evolution and adaptation are not only topics about events happening in the past. We are facing a climatic and temporal catastrophic crisis.

## Supplemental Material

sj-docx-1-jbr-10.1177_07487304231219206 – Supplemental material for Evaluating the Adaptive Fitness of Circadian Clocks and their Evolution Supplemental material, sj-docx-1-jbr-10.1177_07487304231219206 for Evaluating the Adaptive Fitness of Circadian Clocks and their Evolution by Maria Luísa Jabbur, Chitrang Dani, Kamiel Spoelstra, Antony N. Dodd and Carl Hirschie Johnson in Journal of Biological Rhythms
