# Supplementary material for: Evaluating the Adaptive Fitness of Circadian Clocks and their Evolution
Source: J Biol Rhythms. 2024 Jan 7;39(2):115–34. doi: 10.1177/07487304231219206 (PMC10994774; doi:10.1177/07487304231219206)
Supplement: sj-docx-1-jbr-10.1177_07487304231219206 – Supplemental material for Evaluating the Adaptive Fitness of Circadian Clocks and their Evolution [file sj-docx-1-jbr-10.1177_07487304231219206.docx]

**Supplemental Dataset S1**

**Dataset S1:** An AI-based natural language tool does not frame an answer about circadian regulation and fitness in terms of direct evidence for fitness except for a brief mention of “reproductive success” among many other inappropriate indicators. Output is the screenshot from a query to ChatGPT (version 3.5) on 27 April 2023:


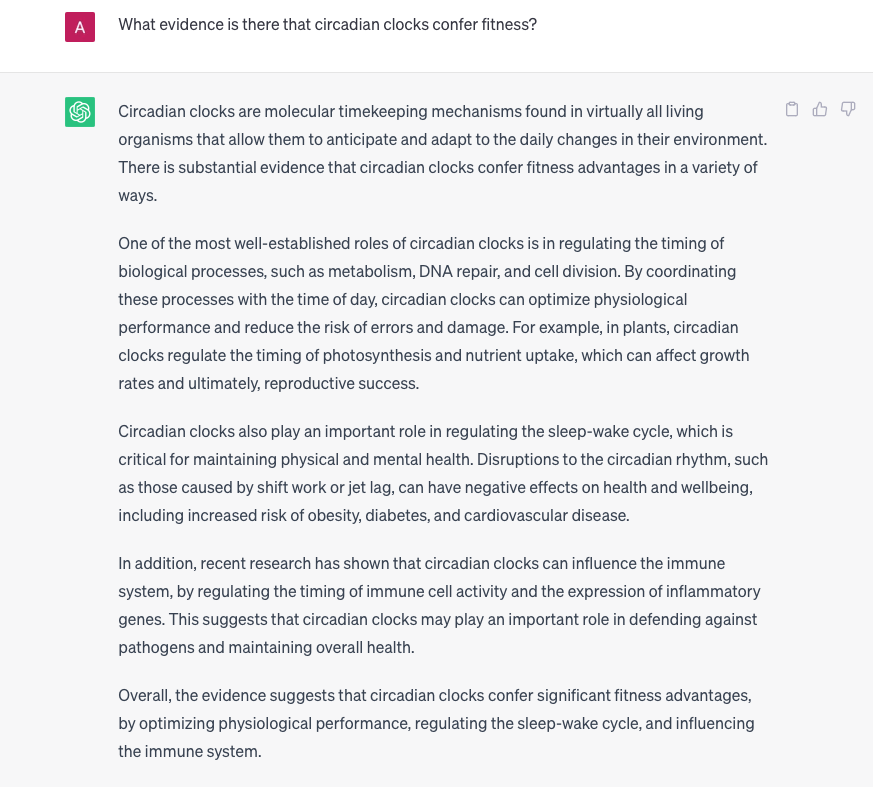


**Acknowledgement**

Supplemental dataset S1 was generated using ChatGPT version 3.5 (https://openai.com/chatgpt).
